# Epigenetic dysregulation of *ID4* predicts disease progression and treatment outcome in myeloid malignancies

**DOI:** 10.1111/jcmm.13073

**Published:** 2017-04-27

**Authors:** Jing‐dong Zhou, Ting‐juan Zhang, Xi‐xi Li, Ji‐chun Ma, Hong Guo, Xiang‐mei Wen, Wei Zhang, Lei Yang, Yang Yan, Jiang Lin, Jun Qian

**Affiliations:** ^1^ Department of Hematology Affiliated People's Hospital of Jiangsu University Zhenjiang Jiangsu China; ^2^ School of Medicine Jiangsu University Zhenjiang Jiangsu China; ^3^ Laboratory Center Affiliated People's Hospital of Jiangsu University Zhenjiang Jiangsu China

**Keywords:** *ID4*, methylation, progression, prognosis, myeloid malignancies

## Abstract

Promoter hypermethylation‐mediated inactivation of *ID4* plays a crucial role in the development of solid tumours. This study aimed to investigate *ID4* methylation and its clinical relevance in myeloid malignancies. *ID4* hypermethylation was associated with higher IPSS scores, but was not an independent prognostic biomarker affecting overall survival (OS) in myelodysplastic syndrome (MDS). However, *ID4* hypermethylation correlated with shorter OS and leukaemia‐free survival (LFS) time and acted as an independent risk factor affecting OS in acute myeloid leukaemia (AML). Moreover, *ID4* methylation was significantly decreased in the follow‐up paired AML patients who achieved complete remission (CR) after induction therapy. Importantly, *ID4* methylation was increased during MDS progression to AML and chronic phase (CP) progression to blast crisis (BC) in chronic myeloid leukaemia (CML). Epigenetic studies showed that *ID4* methylation might be one of the mechanisms silencing *ID4* expression in myeloid leukaemia. Functional studies *in vitro* showed that restoration of *ID4* expression could inhibit cell proliferation and promote apoptosis in both K562 and HL60 cells. These findings indicate that *ID4* acts as a tumour suppressor in myeloid malignancies, and *ID4* methylation is a potential biomarker in predicting disease progression and treatment outcome.

## Background

Myeloid malignancies are a clonal disease derived from myeloid haematopoietic stem/progenitor cells, which usually include MDS, AML and CML. MDS represents a diverse group of clonal haematopoietic disorders characterized by peripheral blood cytopenias, ineffective production of blood cells and high risks of transformation to AML 1. AML is a heterogeneous disease with variable clinical outcome, characterized by the uncontrolled proliferation of granulocytic, monocytic, megakaryocytic or rarely, erythroid blast cells 2. Cytogenetic abnormalities and molecular biological changes including gene mutations and abnormal gene expression play vital roles in leukaemogenesis 3. However, approximately 45% of *de novo* AML is normal karyotypes, whose pathogenesis is complex and remains not well understood, and with a quite heterogeneous clinical outcome from a few days to complete cure 4, 5. CML is a disorder resulted from a reciprocal translocation between chromosome 9 and 22 (known as the Philadelphia chromosome) that codes for *BCR‐ABL* transcripts and fusion proteins with unusual tyrosine‐kinase activity 6. CML is divided into three distinct clinical phases: CP, accelerated phase (AP), and BC according to the course of disease progression 6. Although the molecular pathogenesis of CML is well defined, but the underlying mechanism leading to the progression of CML is not well understood.

Epigenetics refers to variability in gene expression without any underlying modification in the actual genetic sequence, mainly including DNA methylation, histone modifications and microRNAs expression 7. Epigenetic modifications especially DNA methylation play a fundamental role in several aspects of natural development, from embryogenesis taking place in the very early moments after conception, as well as chromatin structure, X chromosome inactivation, genomic imprinting and chromosome stability 7, 8, 9. In addition to the physiological functions, aberrant DNA methylation is also found to be associated with a growing number of human diseases, in particular, human cancers 10. The changes lead to permanent alterations by affecting expression of cancer‐related genes that could regulate the cancer phenotype, such as cellular growth, apoptosis and invasiveness 7. In haematopoietic malignancies, aberrant DNA methylation has been aroused great attentions as crucial molecular events in disease occurrence and progression and also as a predictor for caner progression, diagnosis, risk stratification and prognosis 11, 12, 13.

Highly conserved *ID* (inhibitor of differentiation) gene family (*ID1*‐*ID4*) encodes multifunctional proteins whose transcriptional activity is based on dominant negative inhibition of basic helix‐loop‐helix (bHLH) transcription factors 14. Numerous studies demonstrated an oncogenic function for *ID1*,* ID2* and *ID3* in the initiation and development of cancer including leukaemia 14. In contrast, *ID4* located on a 4 Mb region on chromosome 6p22.3 presents a paradigm shift in context of well‐established role of *ID1*,* ID2* and *ID3* during carcinogenesis 15. Evidence showed that inhibition of *ID4* contributes to developmental defects and cancer progression 11. In a majority of human cancers, *ID4* acted as a tumour suppressor and was low‐expressed caused by its promoter hypermethylation 15. Furthermore, the adverse impact of reduced *ID4* expression on prognosis has been shown in quite a few cancers including colorectal carcinoma, breast cancer and MDS 15. However, *ID4* expression and methylation pattern as well as its direct role in myeloid malignancies were rarely investigated.

In this study, we focused on *ID4* expression and methylation in MDS, AML and CML and further determined *ID4* methylation in predicting prognosis, disease progression and disease surveillance. Moreover, the role of *ID4* in myeloid malignancies was further analysed.

## Materials and methods

### Patients and treatment

This study was approved by Institutional Ethics Committee of the Affiliated People's Hospital of Jiangsu University, and written informed consents were obtained from all participants. A total of 60 healthy donors were used as controls. The diagnosis and classification of 99 MDS, 212 AML and 91 patients with CML were established according to the revised French–American–British (FAB) classification and the 2008 World Health Organization (WHO) criteria 16, 17. The IPSS scores were utilized to classify the risk groups of MDS 18. Our study focused on BM mononuclear cells (BMMNCs) extracted as reported previously 19. The treatment for MDS patients with lower IPSS scores (Low/Int‐1) was symptomatic and supportive treatment with/without thalidomide, whereas patients with higher IPSS scores (Int‐2/High) received chemotherapy included aclacinomycin, cytarabine, granulocyte colony stimulating factor together with symptomatic and supportive treatment. Patients with AML received chemotherapy including induction therapy and subsequent consolidation treatment 20, 21. For non‐M3 patients, induction therapy was one or two courses of daunorubicin combined with cytarabine. Subsequent consolidation treatment included high‐dose cytarabine, mitoxantrone with cytarabine and homoharringtonine combined with cytarabine. Meanwhile, for M3 patients, induction therapy was oral all‐trans retinoic acid (ATRA) together with daunorubicin in combination with cytarabine. Maintenance therapy was oral mercaptopurine, oral methotrexate and oral ATRA over 2 years.

### Gene mutation detection

Gene mutations were detected by high‐resolution melting analysis (HRMA) and direct DNA sequencing as reported 22, 23, 24, 25, 26, 27, 28, 29.

### Cell line and cell culture

Human leukaemic cell lines K562 and HL60 were cultured in RPMI 1640 medium (Thermo Fisher Scientific, Shanghai, China) containing 10% foetal calf serum (ExCell Bio, Shanghai, China) and grown at 37°C in 5% CO_2_ humidified atmosphere.

### Treatment with 5‐aza‐dC

For demethylation studies, cells at a density of 5 × 10^5^ cells/ml in 10 ml were treated with 5‐aza‐dC (Sigma‐Aldrich, Steinheim, Germany) with a final concentration of 0, 1, 2 and 10 μM during 4 days (added daily).

### RNA isolation, reverse transcription and RQ‐PCR

Total RNA isolation and reverse transcription were conducted as reported previously 19. The primers for *ID4* expression were 5′‐CATCCCGCCCAACAAGAAAGTCA‐3′ (forward) and 5′‐GCCGGGTCGGTGTTGAGCGCAGT‐3′ (reverse). *ID4* expression was examined by RQ‐PCR in 7500 Thermo Cycler (Applied Biosystems, Foster, CA, USA) using AceQ qPCR SYBR Green Master Mix (Vazyme Biotech Co., Piscataway, NJ, USA). RQ‐PCR program was carried out at 95°C for 30 sec., followed by 40 cycles at 95°C for 10 sec., 68°C for 1 min., 72°C for 1 min. and 89°C for 30 sec. to collect fluorescence. Relative *ID4* expression was calculated using the following equation:

$$N_{ID4}=2^{▵\mathrm{CT}\;ID4(\mathrm{control}-\mathrm{sample})}÷2^{▵\mathrm{CT}\;ABL(\text{control‐sample})}\left(2^{-▵▵\text{CT}}\right)$$

### DNA isolation, bisulphite modification and RQ‐MSP

Genomic DNA isolation and modification were performed as reported previously 20. RQ‐MSP was applied to detect the level of *ID4* methylation using AceQ qPCR SYBR Green Master Mix (Vazyme Biotech Co., Piscataway, NJ, USA) with primers reported previously 30. RQ‐MSP program was conducted under the conditions: 95°C for 5 min., 40 cycles for 10 sec. at 95°C, 1 min. at 63°C, 1 min. at 72°C and 80°C for 30 sec. The normalized ratio (N_M‐*ID4*_) was used to assess *ID4* methylation level in samples. N_M‐*ID4*_ was calculated using the following formula:

$$N_{M-ID4}=2^{▵\mathrm{CT}\;M-ID4(\mathrm{control}-\mathrm{sample})}÷2^{▵\mathrm{CT}\;ALU(\text{control‐sample})}\left(2^{-▵▵\text{CT}}\right)$$

### BSP

TaKaRa Taq™ Hot Start Version kit (Tokyo, Japan) was used for BSP reaction with primers also as reported 31. BSP conditions were 10 sec. at 98°C, 40 cycles for 10 sec. at 98°C, 30 sec. at 59°C, 30 sec. at 72°C and followed by a final 7 min. at 72°C. BSP products cloning sequencing was performed as described 20, 32. Five independent clones from each specimen were sequenced (BGI Tech Solutions Co., Shanghai, China).

### Western bolt

Western blotting was performed as described previously 33. The antibodies were rabbit anti‐ID4 (Abcam, Cambridge, MA, USA), mouse anti‐β‐actin (Beyotime Biotechnology, Nanjing, China) and antimouse/anti‐rabbit secondary antibodies (Beyotime Biotechnology, Nanjing, China).

### Plasmid construction and transfection

Human full‐length *ID4* CDS sequences cloned in PEX‐2 expression vector (PEX‐2‐*ID4*) were purchased from GenePharma (Shanghai, China). PEX‐2‐*ID4* and PEX‐2 were transfected into K562 and HL60 cells, respectively, using Lipofectamine™ 2000 (Invitrogen, San Diego, CA, USA). *ID4* stably expressed cells were selected by G418 (50 μg/ml; Thermo Fisher Scientific) and flow sorting (BD FACSAriall, San Jose, CA, USA).

### Cell proliferation assays

Cells (1 × 10^5^ cells/ml) were seeded onto a six‐well plate in RPMI 1640 medium containing 10% foetal calf serum. After culturing for 0, 1, 2 and 3 days, cells were counted in counting board for three times.

### Flow cytometry analysis

Cells (2 × 10^5^ cells/ml) were seeded onto a six‐well plate in RPMI 1640 medium containing 1% foetal calf serum (for apoptosis analysis) and 10% foetal calf serum (for cell cycle analysis). Annexin V‐PI apoptosis detection and cell cycle detection kits (BD Pharmingen, San Diego, CA, USA) were used to analyse the apoptosis rate and cell cycle distribution according to the manufacturer's protocols and then analysed *via* flow cytometry (BD FACSCalibur, San Jose, CA, USA). Each experiment was repeated three times.

### TCGA databases

*ID4* methylation (HM450) and mRNA expression (RNA Seq V2 RSEM) data from a cohort of 200 patients with AML from The Cancer Genome Atlas (TCGA) 34 were downloaded *via* cBioPortal (http://www.cbioportal.org) 35, 36.

### Statistical analyses

SPSS 20.0 software package (SPSS, Chicago, IL, USA) was applied to statistical analyses. Mann–Whitney *U*‐test was performed to compare the differences of continuous variables. While, the difference of categorical variables was analysed using Pearson chi‐square analysis/Fisher's exact test. Spearman correlation test was conducted to evaluate the correlation between continuous variables. The ROC curve and area under the ROC curve (AUC) were carried out to assess the discriminative capacity of *ID4* methylation level between patients and controls. Kaplan–Meier and Cox regression (univariate and multivariate) analyses were used to analyse the impact of *ID4* methylation on survival. Statistical significance was set at *P *<* *0.05, and all tests were two sided.

## Results

### Hypermethylation of *ID4* correlated with higher IPSS scores in MDS

**Figure 1:**
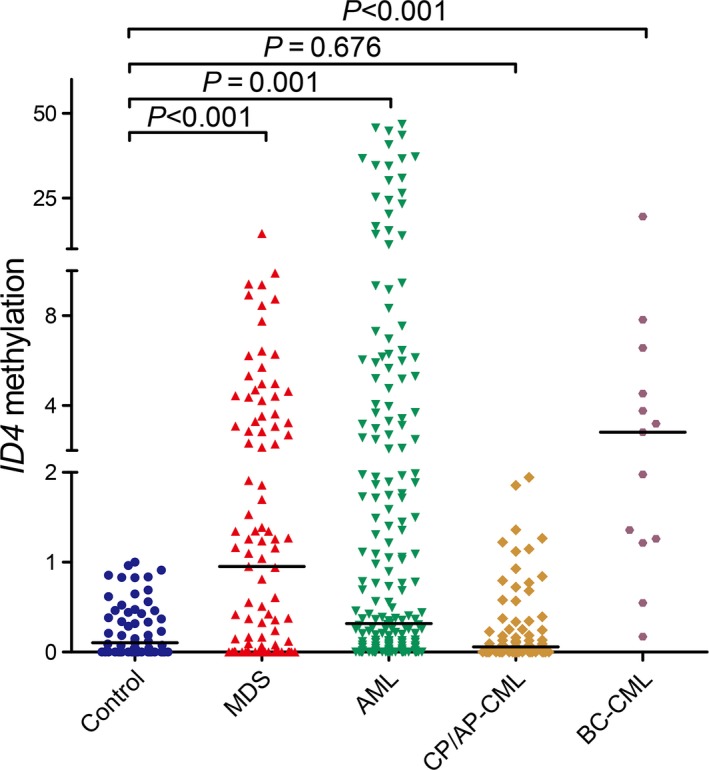
Relative methylation levels of ID4 in controls and myeloid malignancies. The distributions of the *ID4* methylation were presented with scatter plots. The median level of *ID4* methylation in each group was shown with horizontal line.

*ID4* methylation detected by real‐time quantitative methylation‐specific PCR (RQ‐MSP) showed significantly increased level in MDS patients (*P *<* *0.001, Fig. 1). *ID4* methylation was further confirmed by bisulphite sequencing PCR (BSP) in six samples (three controls selected randomly and three patients with highest methylation level). The represented results of BSP were shown in Figure S1. To analyse the correlation between *ID4* methylation and clinical characteristics, patients were divided into two groups (*ID4* hypermethylation and non‐hypermethylation) based on the cut‐off value of 1.021 obtained by receiver operating characteristic (ROC) curve analysis (sensitivity at 49% and specificity at 100%). The comparison of clinical manifestations and laboratory features between the two groups is shown in Table 1. Patients with *ID4* hypermethylation tended to have higher percentage of bone marrow (BM) blasts (*P *=* *0.069). *ID4* hypermethylated patients had higher incidence of *U2AF1* mutation (*P *=* *0.057). Notably, *ID4* hypermethylation occurred significantly in patients with Int‐2/High International Prognostic Scoring System (IPSS) scores compared to those with Int‐1/Low IPSS scores [69% (18/26) *versus* 42% (28/67), *P *=* *0.022].

**Table 1:** Comparison of clinical manifestations and laboratory features between *ID4* non‐hypermethylated and hypermethylated MDS patients

Patient's parameter	Non‐hypermethylated (*n* = 50)	Hypermethylated (*n* = 49)	*P* value
Sex (male/female)	28/22	28/21	1.000
Age (years)	56 (14–85)	62 (20–86)	0.122
WBC (×10^9^/l)	2.9 (1.3–19.5)	2.7 (0.9–82.4)	0.934
HB (g/l)	64 (26–128)	65 (38–118)	0.869
PLT (×10^9^/l)	61.5 (3–1176)	47 (0–754)	0.746
BM blasts (%)	2.0 (0.0–16.5)	6.0 (0.0–27.0)	0.069
Cytogenetic classification			
Good	36 (72%)	34 (69%)	0.677
Intermediate	9 (18%)	7 (14%)	
Poor	2 (4%)	5 (10%)	
No data	3 (6%)	3 (6%)	
IPSS			
Low	7 (14%)	2 (4%)	0.008
Int‐1	32 (64%)	26 (53%)	
Int‐2	8 (16%)	9 (18%)	
High	0 (0%)	9 (18%)	
No data	3 (6%)	3 (6%)	
Gene mutations			
*CEBPA* (+/−)	2/47	0/47	0.495
*IDH1/2* (+/−)	3/46	1/46	0.617
*DNMT3A* (+/−)	0/49	3/44	0.113
*U2AF1* (+/−)	1/48	6/41	0.057
*SF3B1* (+/−)	3/46	3/44	1.000

Median (range); WBC: white blood cells; HB: haemoglobin; PLT: platelet count; BM: bone marrow; IPSS: International Prognostic Scoring System; WHO: World Health Organization; RA: refractory anaemia; RARS: RA with ringed sideroblasts; RCMD: refractory cytopenia with multilineage dysplasia; RCMD‐RS: RCMD with ringed sideroblasts; RAEB: RA with excess of blasts.

### *ID4* methylation was not an independent prognostic biomarker in MDS

**Figure 2:**
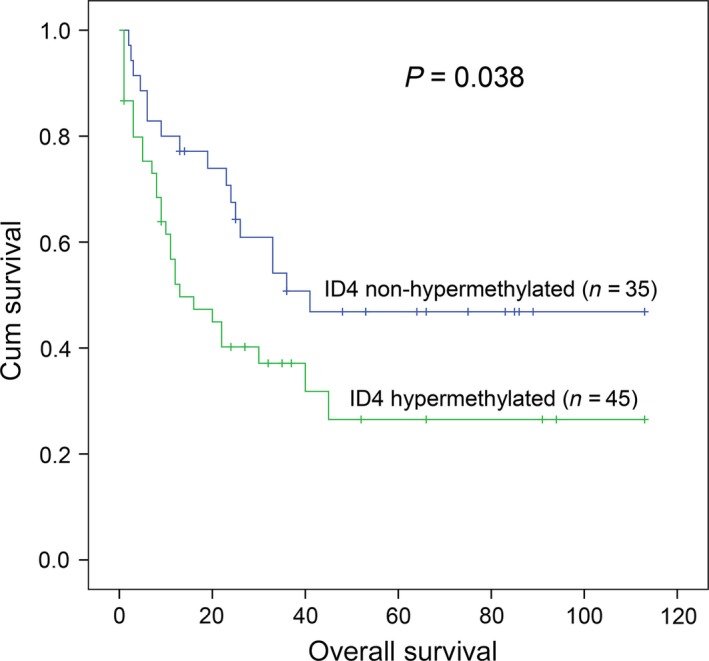
The impact of *ID4* methylation on overall survival (OS) in MDS patients. *ID4* hypermethylated patients showed significantly shorter OS time as compared with ID4 non‐hypermethylated patients which was compared by Kaplan–Meier analysis.

The impact of *ID4* hypermethylation on prognosis was analysed in 80 MDS patients (range 1–113 months; median 26 months). According to Kaplan–Meier analysis, *ID4* hypermethylated patients had a significantly shorter OS time than *ID4* non‐hypermethylated patients (*P *=* *0.038, Fig. 2). However, Cox regression multivariate analysis including variables with *P *<* *0.200 in univariate analysis failed to reveal prognostic value of *ID4* methylation in MDS patients (*P *=* *0.433, Table 2).

**Table 2:** Univariate and multivariate analyses of prognostic factors for overall survival in MDS patients

Prognostic factors	Univariate analyses		Multivariate analyses	
	Hazard ratio (95% CI)	*P* value	Hazard ratio (95% CI)	*P* value
Age	1.867 (1.082–3.222)	0.025	2.420 (1.308–4.477)	0.005
IPSS risks	1.606 (1.141–2.261)	0.007	1.643 (1.107–2.439)	0.014
*ID4* methylation	1.861 (1.018–3.404)	0.044	1.305 (0.670–2.544)	0.433
*CEBPA* mutation	0.406 (0.056–2.949)	0.373	–	–
*IDH1/2* mutation	0.939 (0.293–3.012)	0.915	–	–
*U2AF1* mutation	0.756 (0.272–2.100)	0.591	–	–
*SF3B1* mutation	1.461 (0.454–4.696)	0.525	–	–
*DNMT3A* mutation	2.968 (0.909–9.684)	0.071	2.496 (0.745–8.366)	0.138

IPSS: International Prognostic Scoring System.

Variables including age (≤60 *versus* >60 years old), IPSS scores (Low *versus* Int‐1 *versus* Int‐2 *versus* High), *ID4* methylation (non‐hypermethylated *versus* hypermethylated) and gene mutations (mutant *versus* wild‐type).

### *ID4* methylation was increased during MDS transformed into AML

**Figure 3:**
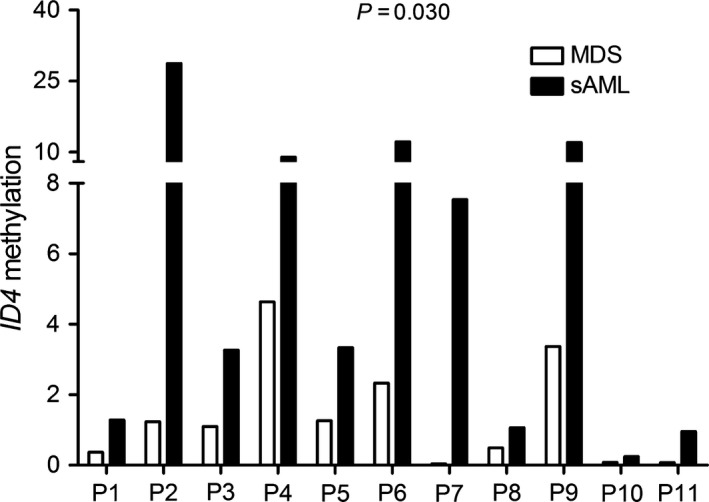
Alterations in *ID4* methylation during MDS to secondary AML (sAML) in 11 follow‐up patients. All patients showed significantly increased *ID4* methylation level in sAML compared to MDS analysed with non‐parametric test.

To verify whether *ID4* methylation was involved in MDS progression, we further determined 11 follow‐up patients with progression from MDS to AML. Of note, *ID4* methylation showed significantly increased in patients with AML (*P *=* *0.030, Fig. 3).

### Hypermethylation of *ID4* was also a frequent event in AML

*ID4* methylation level was also significantly increased in patients with AML (*P *=* *0.001, Fig. 1). *ID4* methylation was further confirmed by BSP in 10 samples (five patients with lowest methylation level and five patients with highest methylation level). The represented results of BSP are shown in Figure S2. *ID4* methylation density in the tested samples was heavily correlated with *ID4* methylation level (*R* = 0.885, *P *<* *0.001). The same cut‐off value also divided the patients into two groups. The comparison of clinical manifestations and laboratory features between the two groups is shown in Table 3. There was a trend that *ID4* hypermethylated patients showed lower platelets (PLT) (*P *=* *0.052). Moreover, the cases with *ID4* hypermethylation had markedly higher white blood cells (WBC) (*P *=* *0.008). The cases with *ID4* hypermethylation presented significantly higher frequency of *CEBPA* mutation (*P *=* *0.026).

**Table 3:** Comparison of clinical manifestations and laboratory features between AML patients with *ID4* non‐hypermethylation and hypermethylation

Patient's parameters	Non‐hypermethylated (*n* = 130)	Hypermethylated (*n* = 82)	*P* value
Sex, male/female	78/52	46/36	0.668
Median age, years (range)	54 (3–87)	50 (17–93)	0.625
Median WBC, ×10^9^/l (range)	10.4 (0.8–528.0)	31.6 (0.3–249.3)	0.008
Median haemoglobin, g/l (range)	74 (32–138)	76 (40–147)	0.187
Median platelets, ×10^9^/l (range)	45 (5–447)	32 (3–264)	0.052
BM blasts, % (range)	48.5 (3.0–97.5)	35.0 (1.0–109.0)	0.147
CR (−/+)	48/47	36/29	0.629
FAB			
M0	0 (0%)	1 (1%)	0.324
M1	12 (9%)	9 (11%)	
M2	50 (38%)	31 (38%)	
M3	18 (14%)	17 (21%)	
M4	26 (20%)	17 (21%)	
M5	17 (13%)	6 (7%)	
M6	7 (5%)	1 (1%)	
Karyotype classification			
Favourable	33 (25%)	25 (30%)	0.846
Intermediate	75 (58%)	46 (56%)	
Poor	16 (12%)	8 (10%)	
No data	6 (5%)	3 (4%)	
Karyotype			
Normal	60 (46%)	32 (39%)	0.599
*t*(8;21)	15 (12%)	7 (9%)	
*t*(15;17)	18 (14%)	17 (21%)	
11q23	1 (1%)	1 (1%)	
Complex	13 (10%)	6 (7%)	
Others	17 (13%)	16 (20%)	
No data	6 (5%)	3 (4%)	
Gene mutation			
*CEBPA* (+/−)	14/111	17/55	0.026
*NPM1* (+/−)	16/109	5/67	0.238
*FLT3*‐ITD (+/−)	17/108	6/66	0.358
*c‐KIT* (+/−)	7/118	2/70	0.491
*N/K‐RAS* (+/−)	9/116	10/62	0.139
*IDH1/2* (+/−)	7/118	5/67	0.761
*DNMT3A* (+/−)	10/115	4/68	0.580
*U2AF1* (+/−)	4/121	3/69	0.708

WBC: white blood cells; FAB: French–American–British classification; AML: acute myeloid leukaemia; CR: complete remission.

### *ID4* methylation was an independent prognostic biomarker in AML

**Figure 4:**
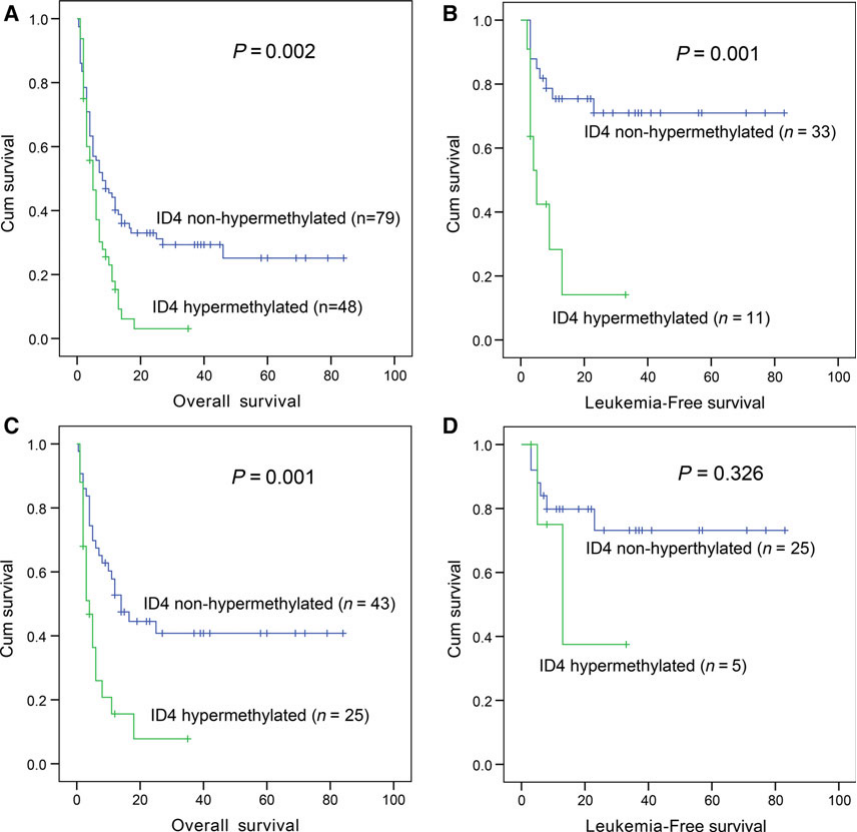
The impact of *ID4* methylation on overall survival (OS) and leukaemia‐free survival (LFS) in patients with AML. (**A**) OS for non‐APL; (**B**) LFS for non‐APL; (**C**) OS for AML with normal cytogenetics (CN‐AML); (**D**) LFS for CN‐AML.

Due to independent disease entity, acute promyelocytic leukaemia (APL) was excluded from the analysis. A total of 127 non‐APL patients with available survival data ranged from 1 to 84 months (median 6 months). Although there was no significant difference in CR rate between the two groups [33% (16/49) *versus* 45% (38/85), *P *=* *0.202], *ID4* hypermethylated cases presented significantly shorter OS and LFS time (*P *=* *0.002 and 0.001, respectively, Fig. 4A and B). Among cytogenetically normal AML (CN‐AML), *ID4* hypermethylation significantly correlated with lower CR rate [30% (8/27) *versus* 57% (27/47), *P *=* *0.030], and shorter OS (*P *=* *0.001, Fig. 4C) but not LFS time (*P *=* *0.326, Fig. 4D). Moreover, Cox regression was further conducted and demonstrated that *ID4* methylation may be act as an independent prognostic factor for OS in non‐APL and CN‐AML patients (*P *=* *0.081 and 0.005, respectively, Table 4).

**Table 4:** Univariate and multivariate analyses of prognostic factors for overall survival in non‐APL and CN‐AML patients

	Non‐APL				CN‐AML			
	Univariate analysis		Multivariate analysis		Univariate analysis		Multivariate analysis	
	HR (95% CI)	*P*	HR (95% CI)	*P*	HR (95% CI)	*P*	HR (95% CI)	*P*
Age	2.229 (1.520–3.269)	<0.001	2.083 (1.364–3.182)	0.001	2.800 (1.544–5.077)	0.001	2.568 (1.345–4.901)	0.004
WBC	1.913 (1.299–2.817)	0.001	1.539 (0.990–2.393)	0.055	1.722 (0.996–3.150)	0.052	1.581 (0.854–2.927)	0.145
Karyotype	1.709 (1.359–2.149)	<0.001	1.675 (1.227–2.288)	0.001	–	–	–	–
*ID4* *	1.845 (1.217–2.798)	0.004	1.507 (0.950–2.391)	0.081	2.695 (1.452–4.999)	0.002	2.483 (1.309–4.712)	0.005
*FLT3*‐ITD†	1.071 (0.558–2.058)	0.836	–	–	0.707 (0.280–1.783)	0.463	–	–
*NPM1* †	1.135 (0.589–2.189)	0.705	–	–	0.967 (0.433–2.159)	0.935	–	‐
*CEBPA* †	0.858 (0.479–1.537)	0.607	–	–	1.075 (0.482–2.394)	0.860	–	–
*c‐KIT* †	0.585 (0.185–1.847)	0.361	–	–	0.404 (0.056–2.927)	0.369	–	–
*N/K‐RAS* †	1.124 (0.583–2.166)	0.727	–	–	1.129 (0.447–2.854)	0.797	–	–
*IDH1/2* †	1.469 (0.783–2.756)	0.230	–	–	1.721 (0.833–3.558)	0.143	1.641 (0.705–3.818)	0.250
*DNMT3A* †	0.948 (0.185–1.847)	0.885	–	–	0.786 (0.312–1.982)	0.609	–	–
*U2AF1* †	2.356 (1.081–5.136)	0.031	2.576 (1.155–5.747)	0.021	2.174 (0.664–7.119)	0.199	1.671 (0.447–6.241)	0.445

*Methylation; ^†^Mutation; HR: hazard ratio.

Variables including age (≤60 *versus* >60 years), WBC (≥30 × 10^9^ *versus* <30 × 10^9^/l), karyotypic classification (favourable *versus* intermediate *versus* poor), *ID4* methylation (non‐hypermethylated *versus* hypermethylated) and gene mutations (mutant *versus* wild‐type).

### *ID4* methylation was decreased in patients with AML achieving CR after induction therapy

**Figure 5:**
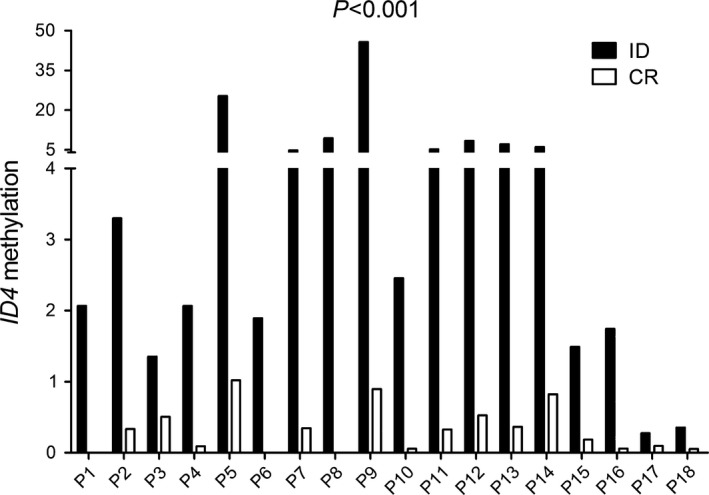
*ID4* methylation changes in patients achieved complete remission (CR) after induction therapy in 18 follow‐up AML. *ID4* hypermethylated patients showed significantly decreased methylation level in CR compared to initial diagnosis (ID) analysed with non‐parametric test.

To identify whether *ID4* methylation could be act as a biomarker for disease surveillance, we assessed *ID4* methylation in 18 follow‐up paired AML patients from the initial diagnosis to CR. Notably, *ID4* methylation was significantly decreased in post‐CR after induction therapy (*P *<* *0.001, Fig. 5).

### Hypermethylation of *ID4* was associated with later clinical stage in CML

*ID4* methylation was further detected in patients with CML and showed significantly hypermethylated in BC stage, but not in AP/CP stage (*P *<* *0.001 and =0.676, respectively, Fig. 1). The patients were also divided into two groups to further analyse its clinical relevance (Table 5). *ID4* hypermethylated cases had significantly lower WBC and PLT (*P *=* *0.017 and 0.041, respectively). According to cytogenetics, patients with *t*(9;22) with additional alteration karyotype had significantly higher frequency of *ID4* hypermethylation compared with patients with *t*(9;22) karyotype [50% (4/8) *versus* 11% (6/54), *P *=* *0.019]. Moreover, the frequency of *ID4* hypermethylation in BC stage was significantly higher than in CP/AP stages [85% (11/13) *versus* 9% (7/78), *P *<* *0.001].

**Table 5:** Comparison of clinical manifestations and laboratory features between CML patients with *ID4* non‐hypermethylation and hypermethylation

Patient's parameters	Non‐hypermethylated (*n* = 73)	Hypermethylated (*n* = 18)	*P* value
Sex, male/female	44/29	11/7	1.000
Median age, years (range)	46 (15–83)	53 (22–75)	0.687
Median WBC, ×10^9^/l (range)	82.2 (2.5–321.9)	23.7 (0.9–142.0)	0.017
Median haemoglobin, g/l (range)	101.5 (47–152)	96.5 (57–119)	0.387
Median platelets, ×10^9^/l (range)	393 (22–1175)	200 (30–939)	0.041
Cytogenetics			
*t*(9;22)	48 (66%)	6 (33%)	0.022
*t*(9;22) with additional alteration	4 (5%)	4 (22%)	
Normal karyotype	3 (4%)	2 (11%)	
No data	18 (25%)	6 (33%)	
Staging			
CP	66 (90%)	5 (28%)	<0.001
AP	5 (7%)	2 (11%)	
BC	2 (3%)	11 (61%)	
*BCR/ABL* transcript, % (range)	278.4 (16.9–100658.0)	89.2 (71.9–1340.4)	0.795

WBC: white blood cells; CP: chronic phase; AP: accelerated phase; BC: blast crisis.

### *ID4* methylation was increased during the progression in CML

**Figure 6:**
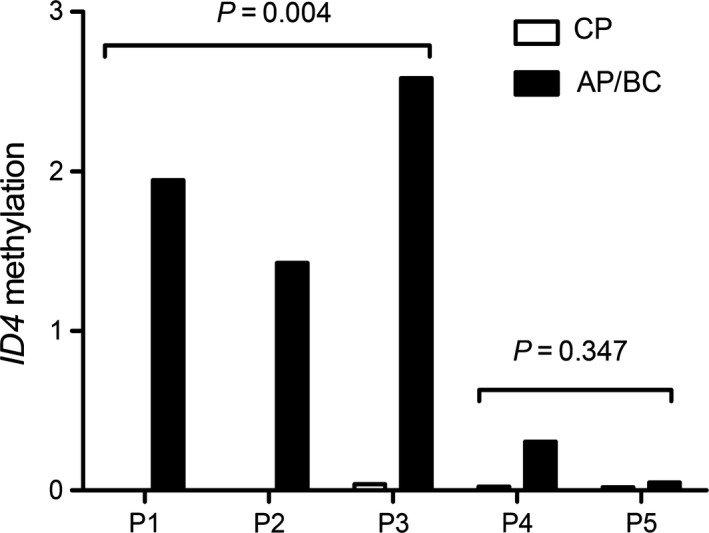
Alterations in *ID4* methylation during CML progression in five follow‐up patients. P1, P2 and P3 were CML in chronic phase (CP‐CML) progression to CML in blast crisis (BC‐CML). P4 and P5 were CP‐CML progression to CML in accelerated phase (AP‐CML). Patients showed significantly increased *ID4* methylation level in CP‐CML progression to BC‐CML but not in CP‐CML progression to AP‐CML.

To confirm that *ID4* methylation was associated with disease progression in CML, *ID4* methylation was further detected in five follow‐up paired CML patents from earlier to later clinical stage. Expectedly, BC‐CML but not AP‐CML showed significantly higher *ID4* methylation level than CP‐CML (*P *=* *0.004 and 0.347, respectively, Fig. 6).

### *ID4* methylation silenced *ID4* expression in myeloid leukaemia

**Figure 7:**
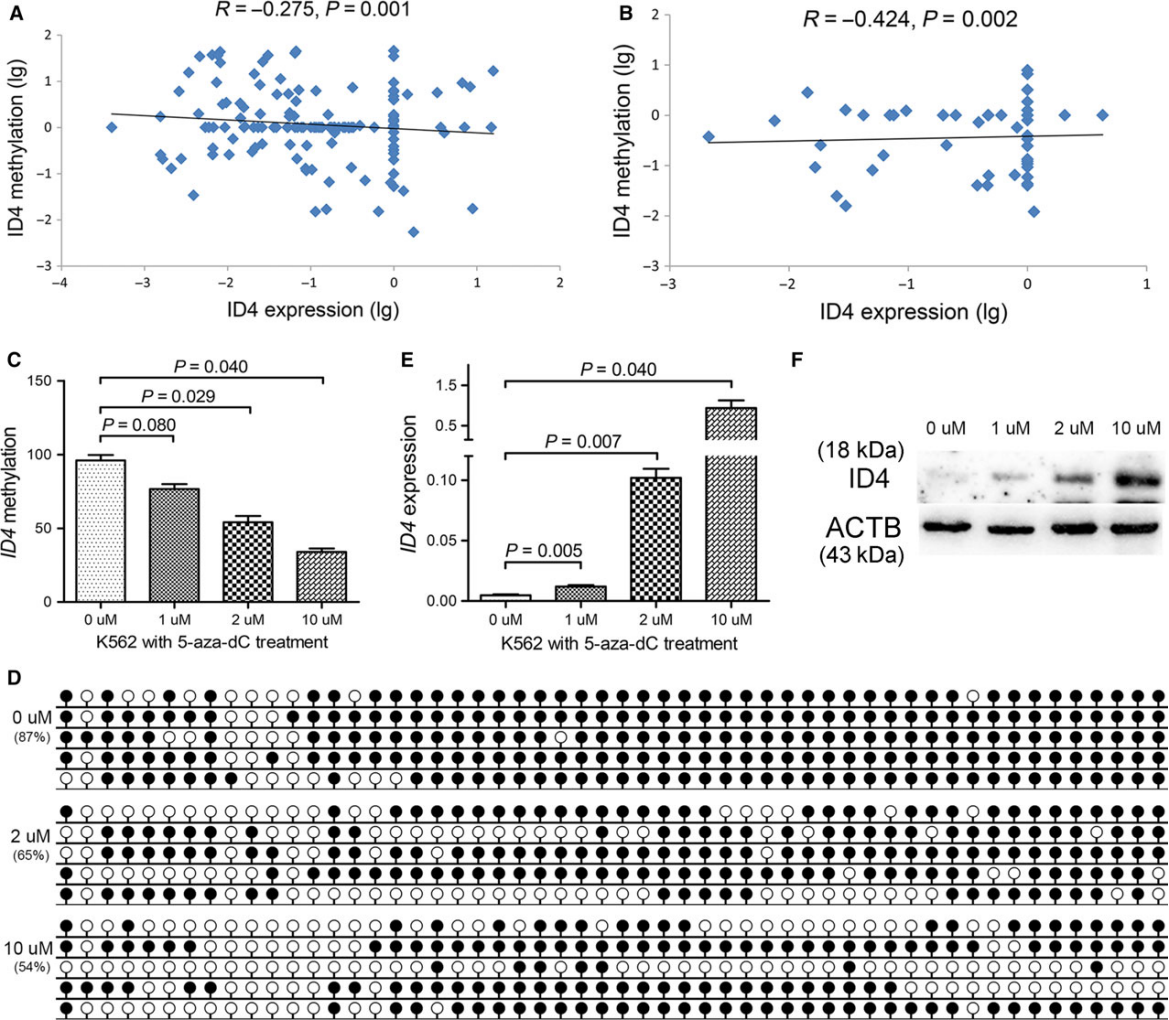
Epigenetic dysregulation silencing *ID4* expression in myeloid leukaemia. (**A** and **B**) correlation between *ID4* methylation and *ID4* expression in patients with CML and AML. (**C** and **D**) *ID4* methylation level and density before and after 5‐aza‐dC treatment. (**E** and **F**) *ID4* transcript and protein level alterations before and after 5‐aza‐dC treatment. White cycle: unmethylated CpG dinucleotide; Black cycle: methylated CpG dinucleotide.

*ID4* transcript level was detected by real‐time quantitative PCR (RQ‐PCR) in 145 AML, 52 CML and 33 control samples with available mRNA. *ID4* expression was significantly down‐regulated in both AML and CML patients (*P *=* *0.003 and 0.006, respectively, Fig. S3). In the tested samples, *ID4* transcript level was negatively correlated with *ID4* methylation level in patients with AML (*R* = −0.275, *P *=* *0.001, Fig. 7A) and patients with CML (*R* = −0.424, *P *=* *0.002, Fig. 7B).

Moreover, an independent assessment of *ID4* methylation and expression in 200 patients with AML from The Cancer Genome Atlas (TCGA) databases also observed a negative correlation between *ID4* methylation and expression (*R* = −0.163, *P *=* *0.034). Moreover, by the median level of *ID4* expression set as the cut‐off value, the cohort of patient with AML was classified into two groups: *ID4* low‐expressed (*ID4* ^low^) and *ID4* high‐expressed (*ID4* ^high^). Although no significant difference was observed in OS time between two groups in non‐APL AML patients, *ID4* ^low^ groups presented markedly shorter OS time than *ID4* ^high^ groups among patients with CN‐AML (Fig. S4).

To explore whether *ID4* promoter methylation could silence *ID4* expression, *ID4*‐hypermethylated K562 cells were treated with 5‐aza‐2′‐deoxycytidine (5‐aza‐dC). As a result, the density of *ID4* methylation was significantly decreased after the treatment (Fig. 7C and D), and *ID4* transcript and protein level was significantly increased (Fig. 7E and F).

### Restoration of *ID4* inhibited cell proliferation and promoted apoptosis

**Figure 8:**
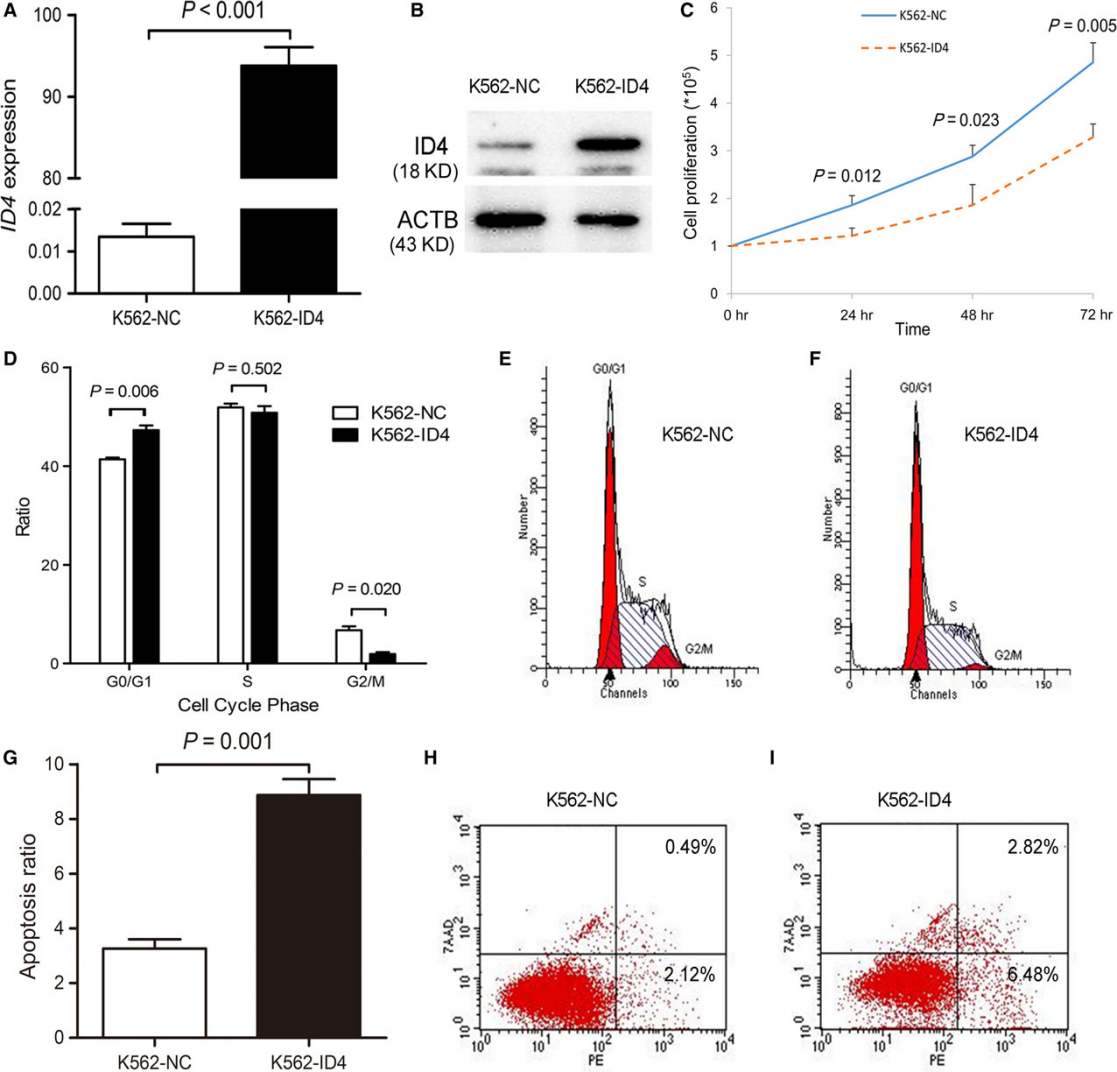
The biological role of *ID4* on leukaemic cell line K562. (**A** and **B**) *ID4* transcript and protein level before and after *ID4* transfection. (**C–I**) the effect of *ID4* on cell proliferation, cell cycle and apoptosis.

**Figure 9:**
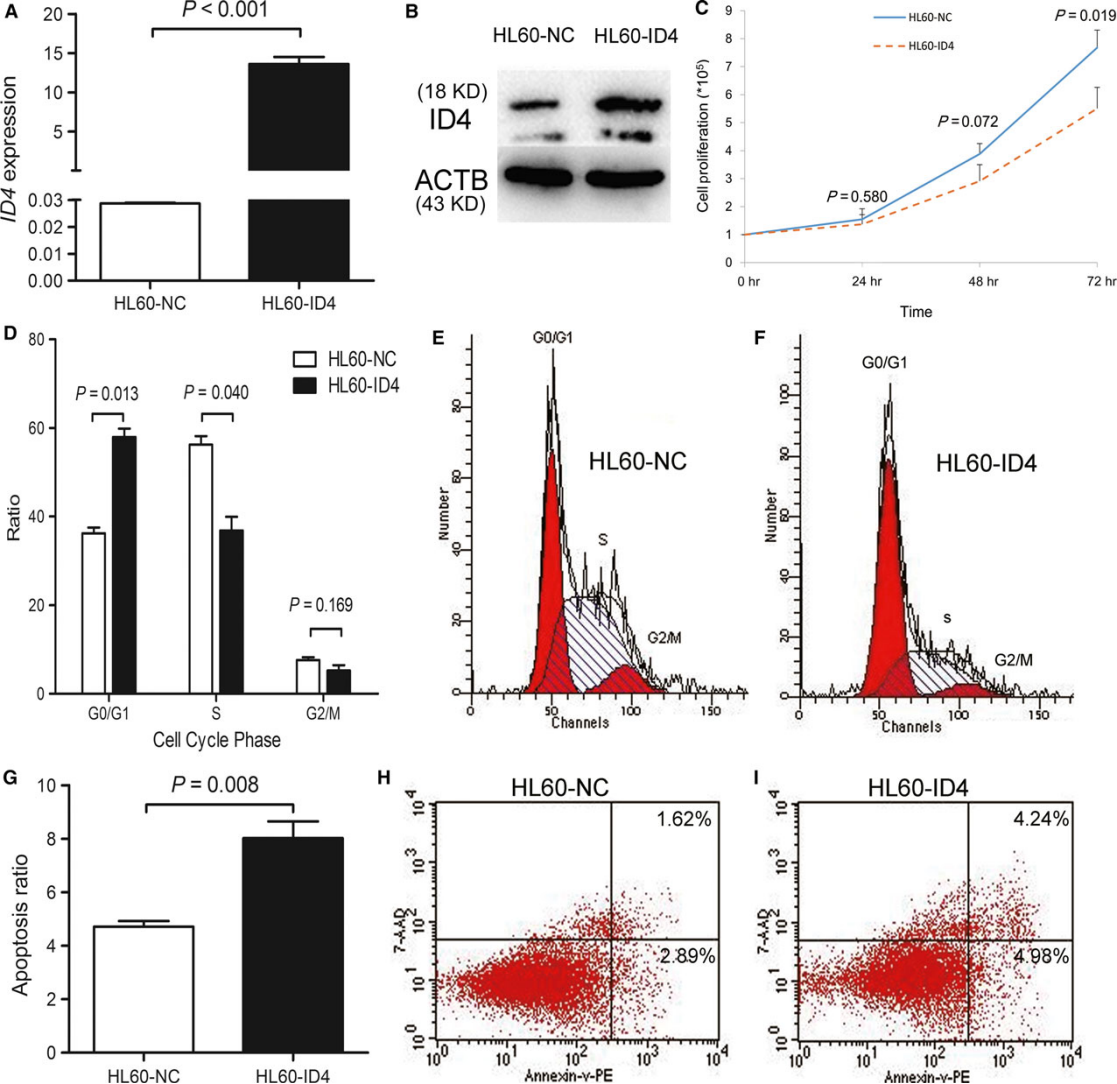
The biological role of *ID4* on leukaemic cell line HL60. (**A** and **B**) *ID4* transcript and protein level before and after *ID4* transfection. (**C–I**) the effect of *ID4* on cell proliferation, cell cycle and apoptosis.

To study the potential biological role of *ID4* in myeloid leukaemia, we performed proliferation assays and apoptosis assays. We established K562 (Fig. 8A and B) and HL60 (Fig. 9A and B) cells overexpressing *ID4* confirmed by RQ‐PCR and Western blot. The proliferation of K562 and HL60 cells was significantly inhibited by *ID4* overexpression (Figs 8C and 9C) and may be caused by G0/G1 arrest (Figs 8D–F and 9D–F). Moreover, an increased ratio of apoptosis was also observed in K562 cells (Fig. 8G,H and I) and HL60 cells (Fig. 9G,H and I) due to *ID4* overexpression.

## Discussion

In the current study, we detected *ID4* methylation in a large cohort of myeloid malignancies using RQ‐MSP, a rapid and precise methodology in detecting DNA methylation 37. Increased *ID4* methylation level was frequently occurred in patients with MDS, AML and BC‐CML. We also confirmed that hypermethylation of *ID4* was one of the epigenetic mechanisms leading to silencing *ID4* expression in clinical samples and leukaemic cell lines. Moreover, by the functional experiments *in vitro*, restoration of *ID4* expression inhibited cell proliferation through cell cycle arrest and promoted cell apoptosis in accordance with previous studies in mouse lymphoma Yac‐1 cells 38. Similarly, ectopic *ID4* expression led to increased apoptosis and decreased cell proliferation due in part by an S‐phase arrest in prostate cancer 39. Chen *et al*. through functional studies *in vivo* revealed that hemizygous loss of *ID4* in non‐transformed TCL1‐positive B cells enhanced cell proliferation triggered by CpG oligonucleotides and decreases sensitivity to dexamethasone‐mediated apoptosis in chronic lymphocytic leukaemia (CLL) 40. In addition, the crossing of *ID4* ^+/−^ mice with Eμ‐TCL1 mice triggered a more aggressive murine CLL 40. These results suggested a crucial role of *ID4* as a tumour suppressor in both lymphoid and myeloid malignancies.

Substantial progress has been achieved in understanding of the underlying mechanism of MDS and CML progression. Chromosomal abnormalities, such as −7/7q‐, +8, 6q‐, 11q‐, *i*(7q), 11q‐, *t*(7;9), *i*(9q) and complex karyotypes (for MDS progression), double Ph chromosome, trisomy chromosome 8, trisomy chromosome 19, *i*(17q), *t*(3;21) and *t*(7;11) (for CML progression), as well as genetic mutations including *TP53*,* DNMT3A*,* TET2*,* IDH1/2*,* EZH2* and *ASXL1* in MDS are considered as progression‐related drivers 41, 42, 43. Recently, epigenetic modifications especially in DNA methylation have been shown contributing to cancer progression including haematological malignancies. Jiang *et al*. reported that aberrant methylation was seen in every sample, on average affecting 91/1505 CpG loci in early MDS and 179 of 1505 loci after blast transformation 11. Our investigation by testing the follow‐up paired patients (MDS to AML and CP/AP‐CML to BC‐CML) indicated that *ID4* methylation was associated with leukaemia transformation in MDS and disease progression in CML. These results together disclosed that *ID4* methylation might also act as vital role contributing to the progression in myeloid malignancies. Accordingly, understanding the mechanisms leading to leukaemic transformation in MDS provides us new antileukaemia therapies.

Clinical implication of *ID4* methylation has been investigated. However, its prognostic impact on prognosis remains controversial in patients with MDS. Previous study by Wang *et al*. suggested that *ID4* methylation was associated with shorter OS or LFS time but not an independent indicator for OS 44. However, a recent study by Kang *et al*. indicated that *ID4* methylation the independently prognostic factor for OS in patients with MDS 45. Our investigation further confirmed the association between *ID4* hypermethylation and adverse prognosis among MDS patients. However, Cox multivariate analysis revealed that it was not an independently prognostic biomarker in MDS patients in accordance with study reported by Wang *et al*. 44. Notably, both Kaplan–Meier and Cox regression analyses disclosed that *ID4* methylation was an independent prognostic biomarker in patients with CN‐AML. Besides this, *ID4* methylation could also act as a promising predictor in disease surveillance in patients with AML. These results together suggested that *ID4* methylation might play a more crucial role in AML. Of course, further studies are needed to determine whether it could be used as a potential predictor for risk stratification in patients with CN‐AML.

Genetic alterations and epigenetic modifications are common molecular events involved in the process of carcinogenesis and interacted with each other. Studies showed that somatic gene mutations such as *IDH1/2*,* DNMT3A*,* TET2*,* ASXL1* and *EZH2* affected epigenetic patterning including DNA methylation and histone modifications in patients with myeloid malignancies 46, 47. In our study, we further investigated the association between *ID4* methylation and common gene mutations in patients with MDS and AML. Interestingly, our data showed that *ID4* methylation was likely to be associated with *U2AF1* mutation in MDS and *CEBPA* mutation in AML. Recently, RNA splicing factors gene *U2AF1* mutation could cause splicing alterations in biological pathways previously implicated in myeloid malignancies, including the DNA damage response and epigenetic regulation usually in DNA methylation through DNMT3B pathway 48. Moreover, our previous study also found that *GPX3* hypermethylation was correlated with *CEBPA* wild‐type in AML, while *DLX4* hypermethylation was associated with *U2AF1* mutation 20, 32. However, the underlying mechanism of the relation between *ID4* methylation *CEBPA* and/or *U2AF1* mutation remains unknown. Further studies are required to determine the role of *ID4* methylation during the leukaemogenesis caused by *CEBPA* and/or *U2AF1* mutation.

Taken together, our study demonstrates that epigenetically silenced *ID4* acts as a tumour suppressor in myeloid malignancies. *ID4* hypermethylation is not an independently prognostic predictor in MDS, but is a valuable indicator in predicting prognosis and disease surveillance in patients with AML. Moreover, *ID4* methylation is associated with disease progression in both MDS and CML.

## Conflict of interest

The authors declared that we have no conflict of interest.

## Supporting information


**Figure S1** Methylation density of *ID4* in controls and MDS patients.

**Figure S2** Methylation density of *ID4* in controls and AML patients.

**Figure S3** Relative expression levels of *ID4* in controls and myeloid leukemia.

**Figure S4** The impact of *ID4* expression on overall survival (OS) in a cohort of 200 AML patients from The Cancer Genome Atlas (TCGA) databases.
