# Supplementary figures and images for: Epigenetic dysregulation of ID4 predicts disease progression and treatment outcome in myeloid malignancies
Source: J Cell Mol Med. 2017 Apr 27;21(8):1468–81. doi: 10.1111/jcmm.13073 (PMC5542913; doi:10.1111/jcmm.13073)

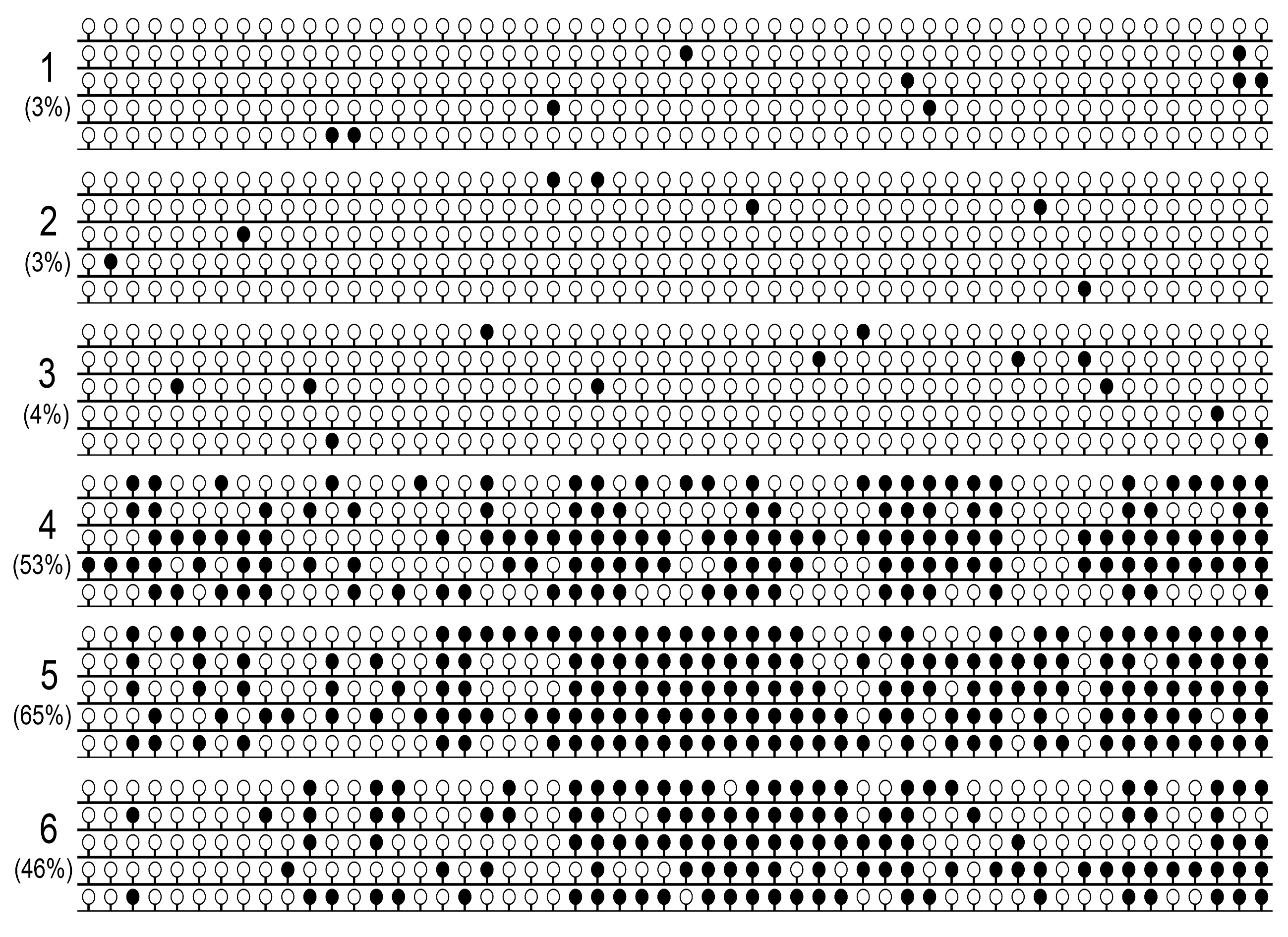

Supplement: Supplementary file 1 — Figure S1 Methylation density of ID4 in controls and MDS patients. [file JCMM-21-1468-s001.tif]

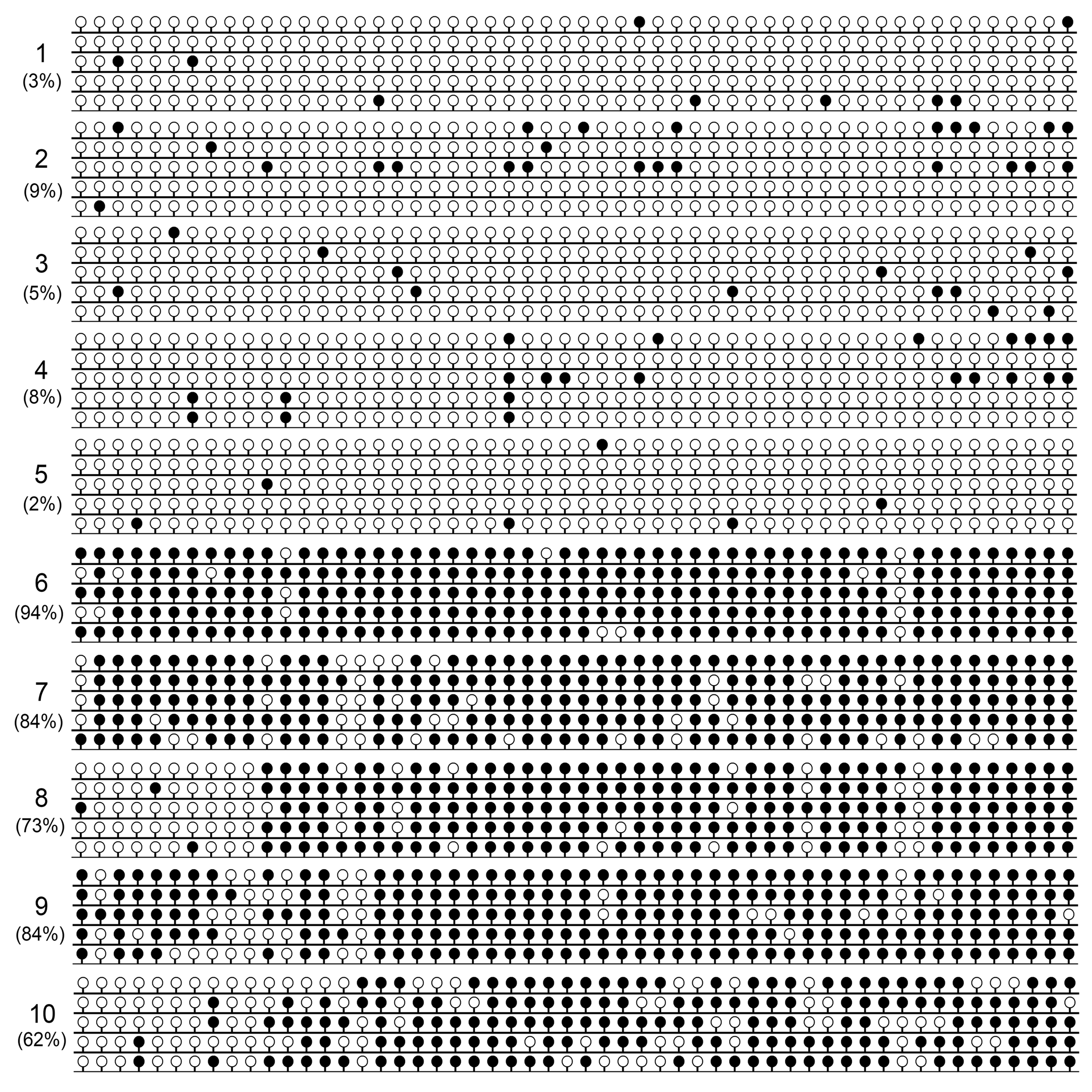

Supplement: Supplementary file 2 — Figure S2 Methylation density of ID4 in controls and AML patients. [file JCMM-21-1468-s002.tif]

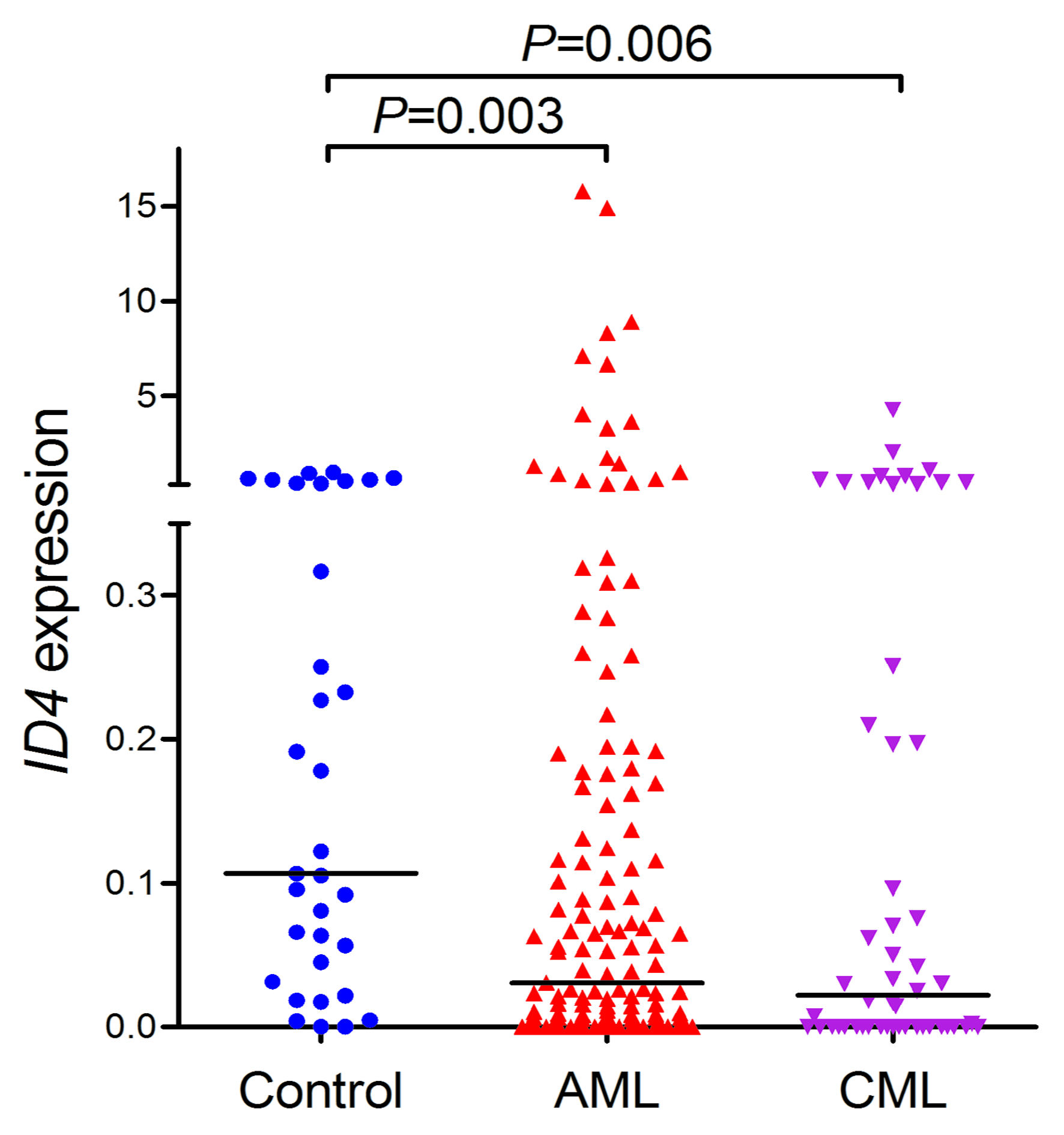

Supplement: Supplementary file 3 — Figure S3 Relative expression levels of ID4 in controls and myeloid leukemia. [file JCMM-21-1468-s003.tif]

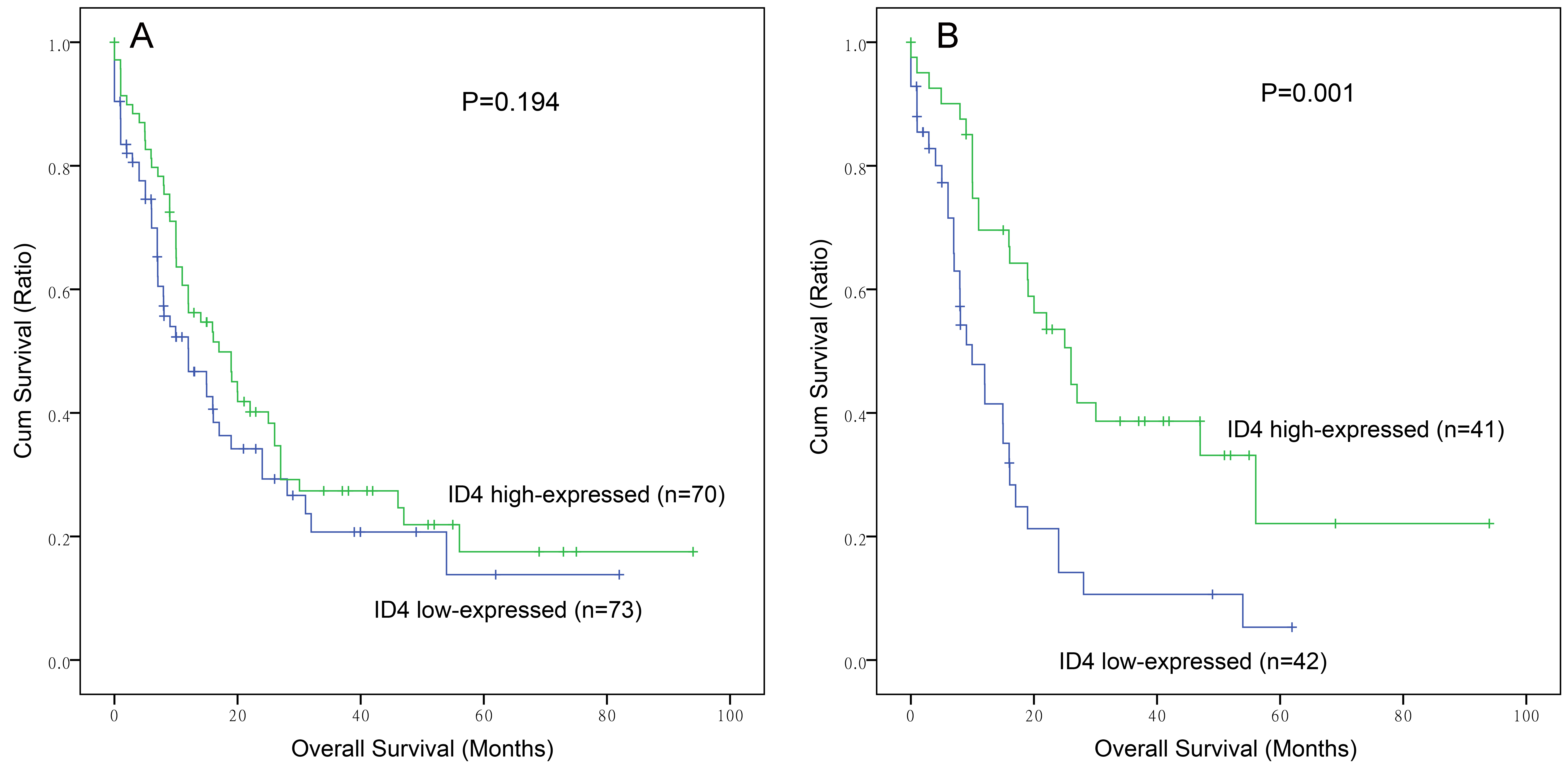

Supplement: Supplementary file 4 — Figure S4 The impact of ID4 expression on overall survival (OS) in a cohort of 200 AML patients from The Cancer Genome Atlas (TCGA) databases. [file JCMM-21-1468-s004.tif]
