# Supplementary material for: Epigenetic dysregulation of ID4 predicts disease progression and treatment outcome in myeloid malignancies
Source: J Cell Mol Med. 2017 Apr 27;21(8):1468–81. doi: 10.1111/jcmm.13073 (PMC5542913; doi:10.1111/jcmm.13073)
Supplement: Supplementary file 5 [file JCMM-21-1468-s005.docx]

**Supplementary Figure 1. Methylation density of *ID4* in controls and MDS patients.** White cycle: unmethylated CpG dinucleotide; Black cycle: methylated CpG dinucleotide. 1-5: three controls selected randomly; 6-10: three MDS patients with highest methylation level selected based on RQ-MSP.

**Supplementary Figure 2. Methylation density of *ID4* in controls and AML patients.** White cycle: unmethylated CpG dinucleotide; Black cycle: methylated CpG dinucleotide. 1-5: five AML patients with lowest methylation level; 6-10: five AML patients with highest methylation level.

**Supplementary Figure 3. Relative expression levels of *ID4* in controls and myeloid leukemia.** The distributions of the *ID4* expression were presented with scatter plots. The median level of *ID4* expression in each group was shown with horizontal line.

**Supplementary Figure 4. The impact of *ID4* expression on overall survival (OS) in a cohort of 200 AML patients from The Cancer Genome Atlas (TCGA) databases.** The patients were classified into *ID4* low-expressed and high-expressed groups by the median level of *ID4* expression. A: OS for non-APL; B: OS for AML with normal cytogenetics (CN-AML).
